# Backpropagation Neural Network-Based Machine Learning Model for Prediction of Blood Urea and Glucose in CKD Patients

**DOI:** 10.1109/JTEHM.2021.3079714

**Published:** 2021-05-13

**Authors:** Jivan Parab, Marlon Sequeira, Madhusudan Lanjewar, Caje Pinto, Gourish Naik

**Affiliations:** Electronics Programme, School of Physical and Applied ScienceGoa University 28961 Taleigao 403206 India

**Keywords:** Artificial Neural Network, chronic kidney, Diabetes mellitus, diabetes nephropathy, Jetson Nano, PLSR

## Abstract

Diabetes mellitus and its complication such as heart disease, stroke, kidney failure, etc. is a serious concern all over the world. Hence, monitoring some important blood parameters non-invasively is of utmost importance, that too with high accuracy. This paper presents an in-house developed system, which will be helpful for diabetes patients with Chronic Kidney Disease (CKD) to monitor blood urea and glucose. This manuscript discusses a comparative study for the prediction of blood urea and glucose using Backpropagation Artificial Neural Network (BP- ANN) and Partial Least Square Regression (PLSR) model. The NVIDIA Jetson Nano board controls the five fixed LED wavelengths in the Near Infrared (NIR) region from $2.0~\mu \text{m}$ to $2.5~\mu \text{m}$ with a constant emission power of 1.2 mW. The spectra for 57 laboratory prepared samples conforming with major blood constituents of the blood sample were recorded. From these samples, 53 spectra were used for training/calibration of the BP-ANN/PLSR model and the remaining 4 samples were used for validating the model. The PLSR model predicts blood urea and glucose with a Root Mean Square Error (RMSE) of 0.88 & 12.01 mg/dL, Coefficient of Determination R^2^ = 0.93 & R^2^ = 0.97, Accuracy of 94.2 % and 90.14 %, respectively. To improve the prediction accuracy, BP-ANN model is applied. Later the Principal Component Analysis (PCA) technique was applied to these 57 spectra values. These PCA values were used to train and validate the BP-ANN model. After applying the BP-ANN model, the prediction of blood urea & glucose improved remarkably, which achieved RMSE of 0.69 mg/dL, R^2^ = 0.96, Accuracy of 95.96 % for urea and RMSE of 2.06 mg/dL, R^2^ = 0.99, and Accuracy of 98.65 % for glucose. The system performance is then evaluated with Bland Altman analysis and Clarke Error Grid Analysis (CEGA).

**Clinical and Translational Impact Statement:** The system designed with Machine learning accurately estimates the Blood Urea and glucose Blood concentration in the samples prepared which conforming to major constituents of human blood tissue. With these encouraging results, the device can be used directly on human cartilage tissue after ethical clearance. This device will immensely help the Diabetes mellitus patient suffering from CKD.

## I. Introduction

The World Health Organization estimates that there are more than 500 million people worldwide are affected by diabetes and is expected to reach 642 million by 2040 [1], [2]. Diabetes is caused by poorly controlled blood glucose levels in the blood, if it remains high (hyperglycemia) for quite a long time, result in the development of serious and life-threatening diseases such as stroke, heart attack, heart failure, kidney failure, adult blindness and amputation [3]. Moreover, many patients also experience episodes of very low blood glucose (hypoglycemia) that can rapidly lead to coma and death [4]. About 40% of people with diabetes will develop chronic kidney disease (CKD) [5].

Diabetes is the leading cause of End-Stage Kidney Disease(ESKD) in most of the developed countries and has driven growth in ESKD globally over recent decades [6]–[8]. There is a strong economic and health imperative to improve outcomes for people with diabetes and kidney disease. In CKD patients continuous monitoring of Blood Glucose and urea is very crucial. Traditionally, CKD believed to result from diabetes has been termed “diabetic nephropathy.” Recently, the Diabetes and CKD workgroup of the National Kidney Foundation Kidney Disease Outcomes Quality Initiative (KDOQI) suggested that a diagnosis of CKD presumed to be caused by diabetes should be referred to as “Diabetic Kidney Disease (DKD)” and the term “diabetic nephropathy” should be reserved for kidney disease attributed to diabetes with histopathological injury demonstrated by renal biopsy [9]. Kidney damage may be demonstrated by abnormal imaging studies, urine sediment, urine chemistries, or, more commonly, proteinuria, blood urea nitrogen [10].

Monitoring blood urea and HbA1c are problematic in CKD due to reduced red cell survival time, use of erythropoietin, modifications of hemoglobin (e.g., carbamylation), and mechanical destruction of red blood cells on dialysis. Thus, clinicians may often need to rely more on random or continuous home blood glucose and urea monitoring. This is a tedious, inconvenient approach in people with CKD, who are often sick and frail [11].

Till now, a cure for diabetes has not been found, researchers are trying to develop an effective glucose monitoring device that will reduce complications associated to a certain extent [12]. There are several pick-based glucose monitors, which are based on the electrochemical principle [13]. This invasive testing not only causes pain but also there are high chances of infection risk. This is the reason why a patient with diabetes is hesitant to monitor glucose several times a day as recommended over the years [14], [15]. Hence the time demands the non-invasive approach for monitoring blood glucose. Non-invasive glucose sensing is yet another measurement strategy that promises pain-free operation without the complications of an adverse biological response [16], [17]. Various spectroscopic methods for non-invasive estimation of blood glucose have been proposed, such as Raman, fluorescence, and bioimpedance spectroscopies, as well as polarimetric, photonic crystal, optoacoustic, optothermal, and optical coherence tomography [18]. We have aimed to use NIR spectroscopy since glucose, urea, and other blood constituents have better signature in this region, and also the absorptivity of water absorptivity is minimal.

In the last few years, the use of Artificial Neural Network (ANN) has been used more often for qualitative and quantitative analysis because of its advantages such as anti-interference, anti-noise, and strong nonlinear transmission capability. ANN is a new technique of information processing that is based on neuroscience research and is created by simplifying and simulating biological structure. ANN possesses some important features such as it can approximate any complex nonlinear relations; robustness and fault tolerance; it can emulate and adapt to the unknown system and at the same time can deal with the quantitative and qualitative knowledge [19], [20]. Among all, the most famous and widely used is the BP-ANN model. BP-ANN model realizes highly nonlinear mapping between input and output, this model can achieve any continuous nonlinear curve. The BP-ANN is preferred over others because of its capability in handling both linear and nonlinear relationships [21].

## II. Wavelength Selection and Sample Preparation

### A. Selection of Wavelength Region

**FIGURE 1.**
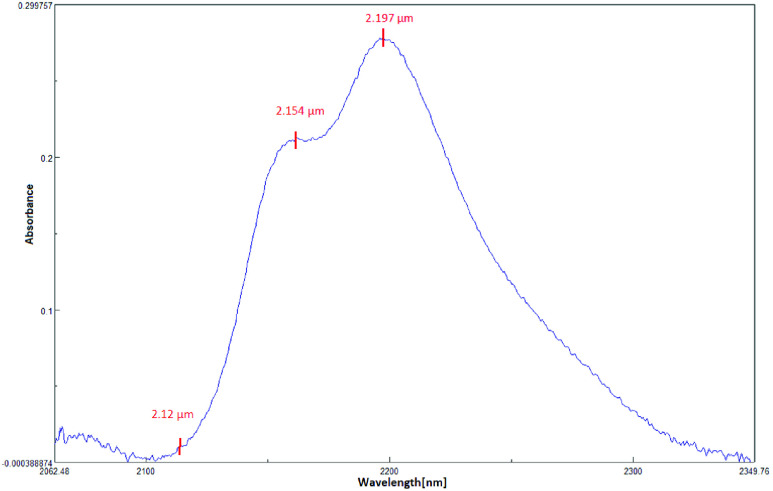
Spectra of urea recorded with Jasco 770.

**FIGURE 2.**
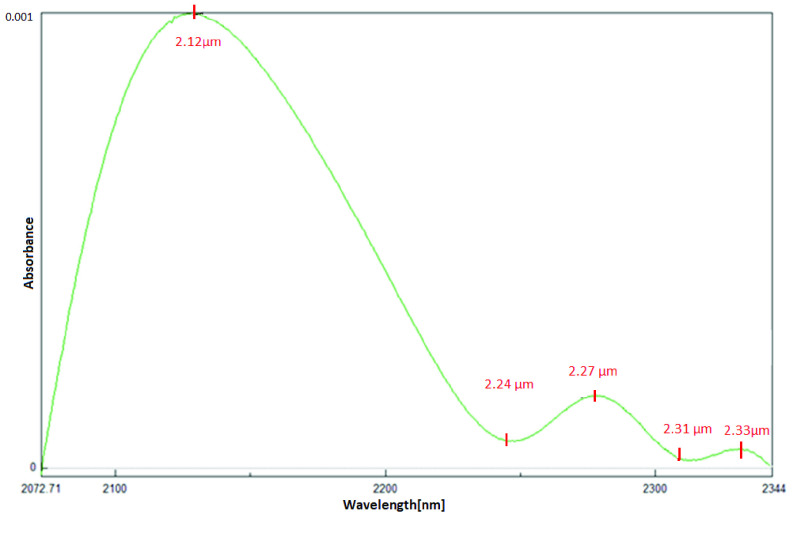
Spectra of glucose recorded with Jasco V770.

Infrared spectroscopy is based on optical absorption and scattering of infrared radiation when impinges on human tissue due to its interaction with biological components within the tissues. This includes both Mid Infra-Red (MIR) and NIR spectroscopies. Though the wavelength range suggested in NIR for this region vary in the standards, we used $0.7\mu \text{m}$ to $2.5\mu \text{m}$ for NIR and $2.5\mu \text{m}$ to $25\mu \text{m}$ for MIR [22]. The NIR spectra are mostly made up of broad bands corresponding to overlapping peaks and first, second, third and combination overtones formed by molecular vibrations. This technique is based on the variations in radiation intensity caused due to transmittance and reflectance [23]–[25]. The probing of human tissue is preferred in the NIR region as the water component present in human tissue has a good transmission window. Also, InGaAs detectors are comparatively cheaper than to HgCdTe detector. Also, glucose and urea exhibit significant signature peaks in the NIR region. The above reasons motivate the development of a sensor-based NIR absorption spectroscopy. The absorbance peaks and valleys of urea and glucose in the NIR range from $2\mu \text{m}$ – $2.5\mu \text{m}$ are shown in Figure 1 & Figure 2 respectively.

Our system uses NIR LEDs at the wavelength in the vicinity of the peaks and valleys of urea and glucose namely $2.12~\mu \text{m}$, $2.24~\mu \text{m}$, $2.27~\mu \text{m}$, $2.31~\mu \text{m}$ & $2.33~\mu \text{m}$. Traditionally, NIR spectroscopy relied upon using a white light source to probe urea and glucose, which makes the whole equipment bulky and power-hungry. By making use of LEDs, we are stepping towards making the entire system portable and affordable.

### B. Data Preparation for Prediction Model

**FIGURE 3.**
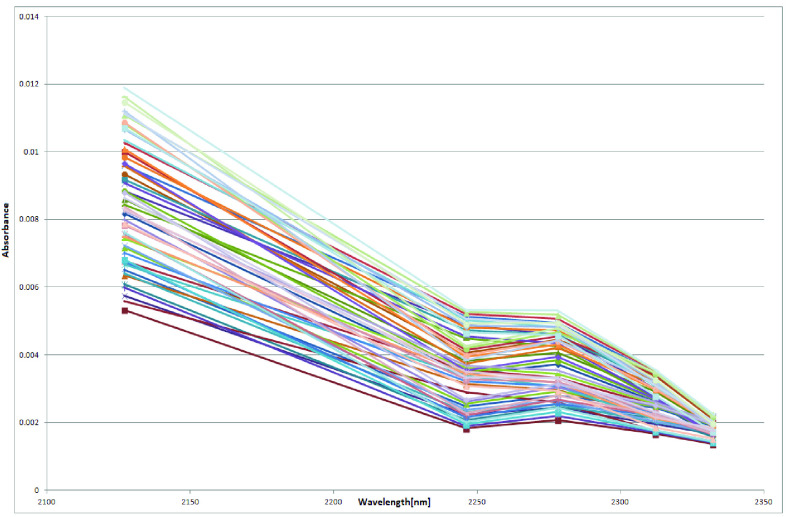
Spectra’s of 57 laboratory samples.

57- Laboratory samples were prepared which resembles the blood by mixing Glucose, ascorbate, urea, lactate, and alanine in the proportion found in the blood in an aqueous solution. The Analytical Grade compounds were procured from Sigma Aldrich Ltd. The spectra of all 57 different aqueous samples by mixing the above 5 constituents in different proportions as shown in figure 3. Table 1 shows typical 10 of these samples absorbances at 5 selected wavelengths. We have used a 1mm pathlength Quartz glass cuvette to reduce water absorbance effects and thus increases the S/N ratio.

**TABLE 1:** 10 Typical Samples

Sr No.	Concentration in mg/dL					Absorbance at the wavelength($\mu \text{m}$) $\times 10^{-3}$				
	Urea	Lactate	Ascorbate	Alanine	Glucose	2.33	2.31	2.27	2.24	2.12
1	11	12	2	10	70	1.59	2.62	3.72	3.41	8.17
2	11	22	5	10	70	2.12	3.38	4.44	4.07	9.35
3	20	22	2	10	70	1.91	3.17	4.60	4.73	9.19
4	11	12	2	28	100	1.42	1.72	2.20	1.90	5.99
5	11	22	5	28	100	1.94	2.48	2.91	2.56	7.17
6	20	22	5	10	100	2.25	3.51	5.07	5.21	10.27
7	11	22	2	28	200	1.65	2.21	2.68	2.21	7.58
8	20	12	5	28	200	1.81	2.13	3.18	3.44	7.49
9	11	12	2	28	280	1.45	1.77	2.43	2.02	7.62
10	20	12	2	10	280	1.75	2.80	4.58	4.67	10.71

If one looks at the absorbance at these LED wavelengths as given in above table 1. It is observed that as the wavelength is decremental from $2.33~\mu \text{m}$ to $2.12~\mu \text{m}$ they follow an incremental pattern in the absorption when the urea concentration is maximum i.e. 20 mg/dL. When urea is minimum (11 mg/dL), the absorbance was found to be minimum at $2.24\mu \text{m}$ unlike for a sample of urea concentration i.e 20 mg/dL. Which clearly indicates the influence of urea concentration on the prediction of glucose. Hence, in order to predict urea and glucose, we require to train two different models one each for urea and glucose.

## III. Methodology

The entire system is designed using NVIDIA JETSON Nano Board having ARM Cortex-57 which triggers individual NIR LED sources (2.12, 2.24, 2.27, 2.31 & $2.33\mu \text{m}$). The LED’s with their drivers and detector was procured from IBSG Co. Ltd Company (Russia). The LEDs used have been standardized by appropriate current limiting resistance so that the output power is 1.2 mW. The individual LED radiation is then allowed to pass through the sample placed in a 1mm cuvette. The light intensity passing through the sample is attenuated according to the beer lamberts law in proportion to the sample concentration. The detected light by the PD24-20 (InGaAs detector) having responsivity in 1.15 – 2.40 $\mu \text{m}$ is then conditioned with a gain of 100 and given to A/D converter ADS1015 which has a resolution of 12 bit.

**FIGURE 4.**
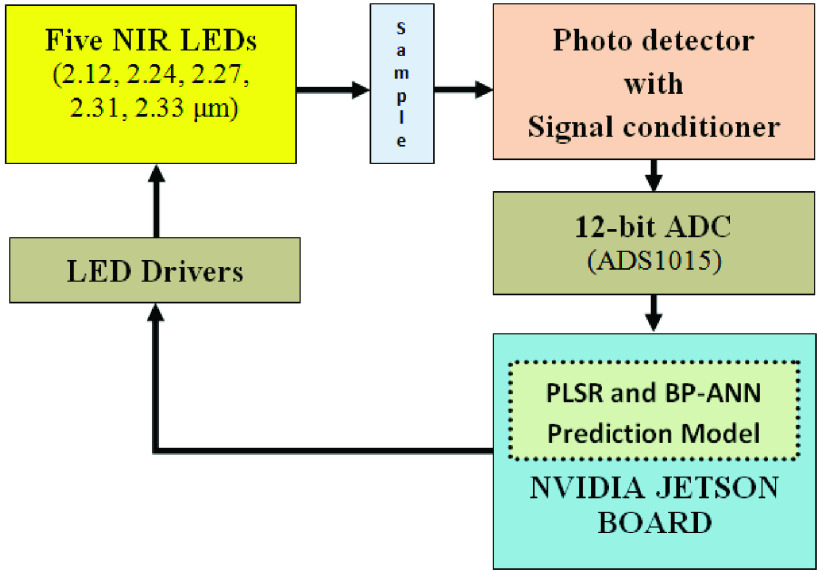
Block diagram for estimation of blood urea and glucose.

The block of the entire system is shown in figure 4. The principles of acquiring the signal are based on Beer-Lambert Law, which relates the incident power to the concentration of the absorbing sample and also to the path length.

\begin{equation*} A=-{log}_{10}\left ({\frac {P}{Po} }\right)=a\times b\times c\tag{1}\end{equation*}

where “A” is absorbance, “P” is the radiant power, absorptivity is denoted by “a”, the path length is denoted by “b”, and concentration is denoted by “c”.

**FIGURE 5.**
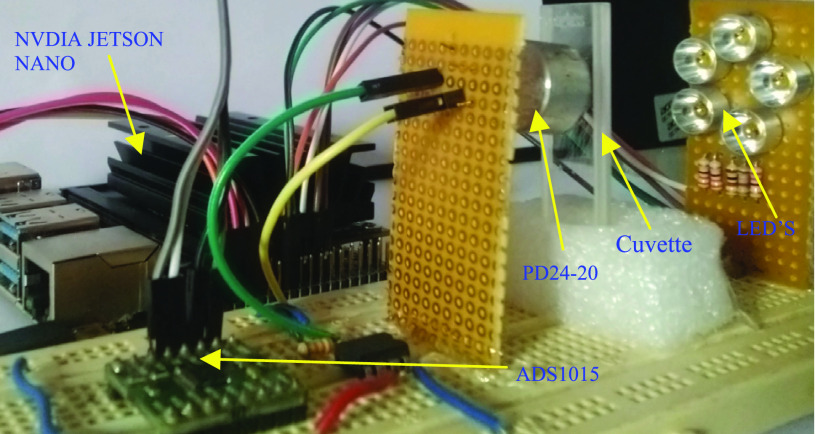
The Photo of the entire setup.

The LEDs corresponding to the particular wavelength are sequentially powered on, and the corresponding readings are recorded. The absorbance is calculated using equation (1). This is done for all the 57 laboratory prepared samples. These readings are arranged in two datasets with 53 in calibration/ training and 4 in prediction. On these datasets, Python code for PLSR and BP-ANN is executed on NVIDIA Jetson Nano to estimate the blood urea and glucose in 3–5seconds. Figure 5 shows the photo of the entire setup. Python code for PLSR and BP-ANN is executed on NVIDIA Jetson Nano to estimate the blood glucose and urea in 3–5 seconds.

### A. Partial Least Square Regression (PLSR)

PLSR is an extension of the multiple linear regression(MLR) models. In its simplest form, a linear model specifies the relationship between a dependent variable “$Y$” and a set of predictor variables, the “*X”.* The PLSR finds its use in the area of NIR non-destructive estimation of biomolecules. As PLSR is a linear model which doesn’t consider nonlinear characteristics of data while predicting [26]. This model works very well if there are a large number of spectral data points. As we reduce the spectral points, the prediction error suffers. Hence to improve the RMSE and accuracy an ANN model namely BP-ANN is used.

### B. Backpropagation Artificial Neural Network (BP-ANN)

**FIGURE 6.**
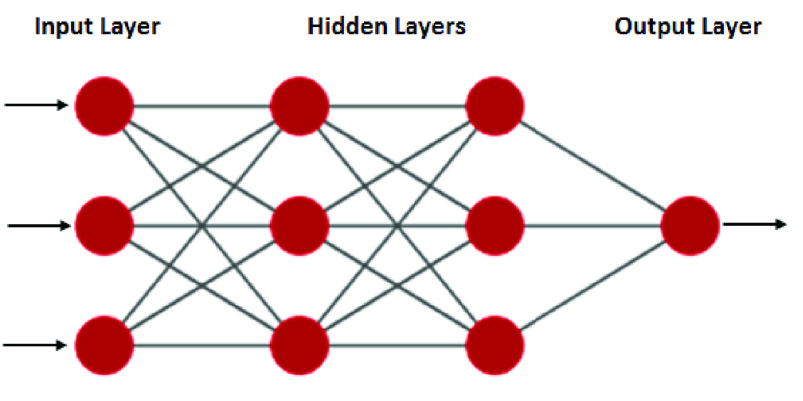
A-4-layer BP-ANN architecture for urea.

Here, we have used feed-forward neural network architecture. This network is trained using the Back propagation (BP) algorithm which is a form of supervised learning of a multi-layer network. Error at the output layer is back propagated to earlier ones to update weights and bias of these layers. We have implemented two BP-ANN models one for urea and the other for glucose estimation. Training in the sequential mode is advantageous because it is stochastic in nature and hence can avoid local minima [27]. BP-ANN model for urea has two hidden layers with 3 neurons each, 3 neurons at the input layer, and one neuron in the output layer. In this network, we will be predicting urea values based on the three Principal component Analysis (PCA) inputs. The input data set (X) is a $53\times 3$ matrix of three PCA components and output data (Y) is a $53\times 1$ matrix of urea values. The Four-layer BP-ANN architecture is shown in figure 6.

**FIGURE 7.**
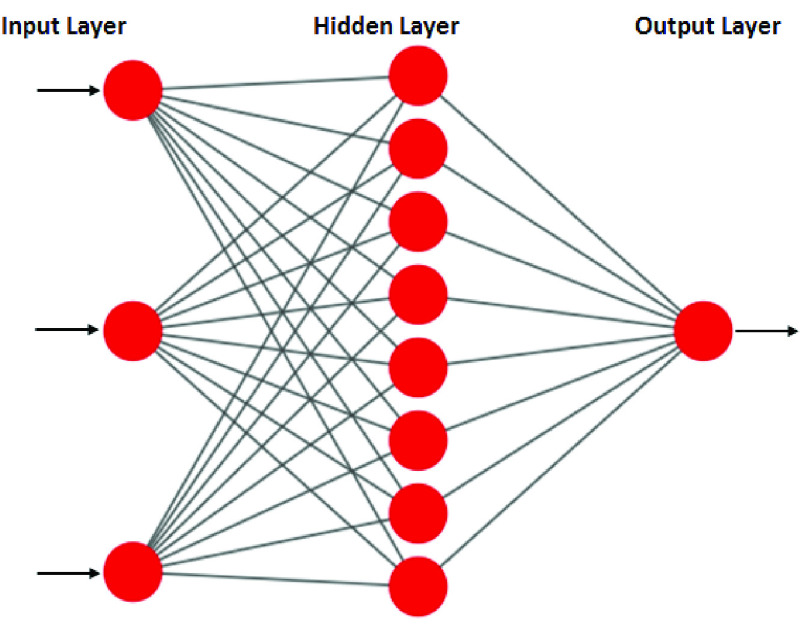
A-3-layer BP-ANN architecture for glucose.

Whereas the BP-ANN model for glucose prediction has one hidden layer with 8 neurons, 3 neurons at the input layer, and one neuron in the output layer Here also, PCA inputs are given to the input layer. The three-layer BP-ANN architecture is shown in figure 7. The BP-ANN algorithm is implemented on Jetson Nano using Python programming language.

**FIGURE 8.**
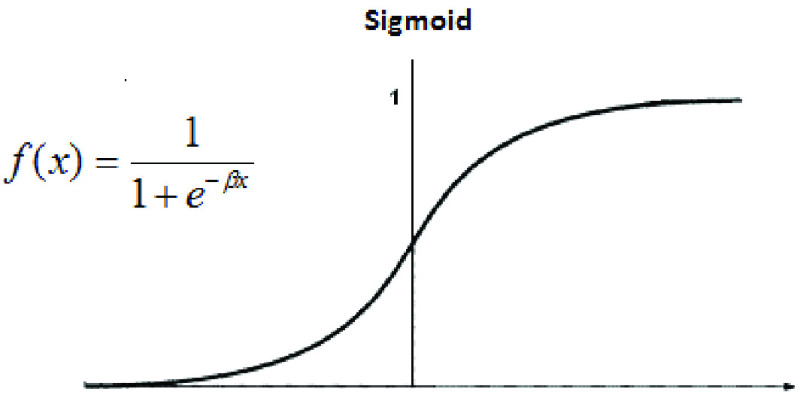
Activation function.

First, the network is trained with $53\times3$ inputs and $53\times1$ corresponding outputs. Each element of input X is multiplied by a corresponding weight and then added together with all the other results for each neuron in the hidden layer. The sigmoid activation function used for both models as shown in figure 8 is used to get the final value for the hidden layer.

**FIGURE 9.**
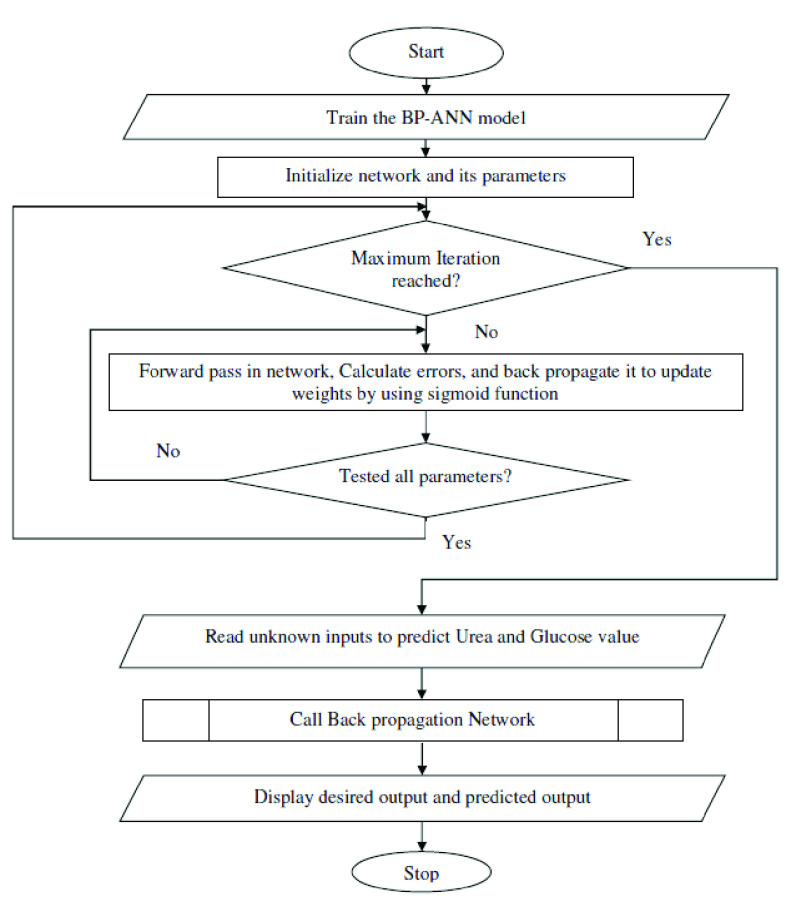
Flowchart of BP-ANN for urea and glucose estimation.

The flowchart of the BP-ANN for urea and glucose estimation is shown in figure 9. In the BP-ANN algorithm, spectral data is procedurally divided into two subsets: the first one is called a calibration or training set and the other is a prediction or validation set. The training data are used to calibrate/train the network and a validation set is used to check the prediction accuracy of the network.

## IV. Results & Discussion

### A. Estimation of Urea and Glucose Prediction

Here, we have developed PLSR and BP-ANN model for estimating urea and glucose.

PLSR technique is commonly used for analysis in econometrics and social sciences and is an excellent method for the determination of the concentration of blood analytes, such as glucose, ascorbate, lactate, urea, cholesterol, etc. For applying this technique to predict glucose concentration, a set of calibration training data is formulated from the collected absorption spectra of five major constituents which contain absorption signatures associated with glucose along with the absorption of other constituents [28].

In the PLSR method, both the absorption data and the concentration data are used at the outset to formulate a calibration model. The PLSR algorithm is developed using Python programming language and ported on Jetson Nano to predict the urea and glucose concentration.

**FIGURE 10.**
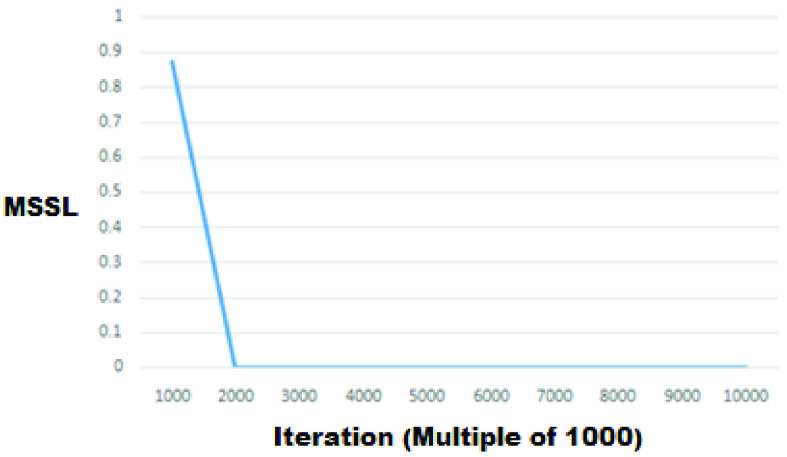
MSSL vs Iterations for Urea.

**FIGURE 11.**
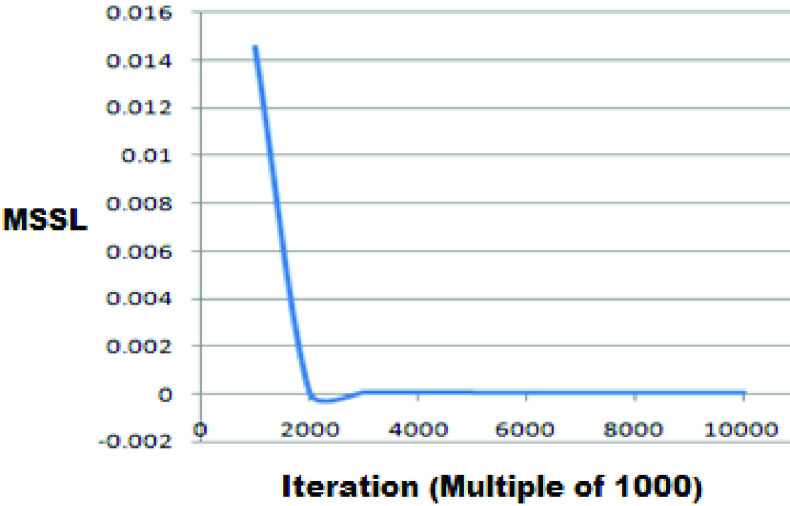
MSSL vs Iterations for Glucose.

A 4-layer & 3-layer BP-ANN is implemented on the Jetson platform. Here, the important step is to train the network. To get the best results Mean Sum Squared Loss (MSSL) should be less than 0.002 which is achieved at the $2000^{\mathrm {th}}$ iteration for urea as seen from figure 10. For glucose, MSSL is shown in figure 11. Further increase in the number of iterations further reduces the MSSL. We have run the model for 10000 iterations to observe any significant change in MSSL. A large data sample for training means the output of the system will be more accurate. In order to eliminate redundant interference, we have not trained the network with original spectral data but we have performed PCA on these data. Then the optimal principal component (PC) PC1, PC2 & PC3 are selected as the inputs for the BP-ANN model.

After completion of network training, we have to test the network for the prediction of urea and glucose concentration. The prediction result for urea and glucose with both PLSR & BP-ANN models is depicted in table 2 and 3 respectively. From table 2 & 3, it is very clear that the best estimation for both is achieved with BP-ANN. The estimated/predicted values are very near to the actual concentration whereas prediction with the PLSR model has a large error.

**TABLE 2:** Urea Predicted Result

Actual Urea (mg/dL)	Estimated Urea (mg/dL)in PLSR Model	Predicted Urea (mg/dL) in BP-ANN Model
11	11.54	11.25
20	19.41	19.66
16	17.20	17.14
13	11.98	12.34

**TABLE 3:** Glucose Predicted Result

Actual Glucose (mg/dL)	Estimated Glucose (mg/dL) in PLSR Model	Predicted Glucose (mg/dL) in BP-ANN Model
70	57.91	70.45
100	120.53	103.79
200	198.73	201.39
280	277.21	280.71

The predicted values depending on how the model is fitted to the calibration set. Hence, in some cases, the predicted values are higher than the actual, and in some, it is lower. The RMSE for estimating blood urea with PLSR Model is 0.88 mg/dL and with BP-ANN Model is 0.69 mg/dL. And also, the RMSE for estimating blood glucose with PLSR Model is 12.01 mg/dL and with BP-ANN Model is 2.06 mg/dL.

### B. System Validation

Here, we have used Bland –Altman analysis, coefficient of determination (R^2^) for urea, and Glucose for validation. Clark Error Grid Analysis (CEGA) was used to evaluate the agreement between the estimated and actual values of Blood Glucose.

#### 1) Bland-Altman Analysis

The Bland –Altman analysis is used to compare the estimated and actual values graphically. It is a scatter plot of the difference between the estimated and the actual reading on the Y-axis and the corresponding mean of the estimated and actual values on the X-axis [29]. Horizontal lines are drawn at the mean of the difference and at the limits of agreement, which are defined ±1.96 times the Standard Deviation (SD) from the mean of difference.

\begin{equation*} \mathrm {SD=}\sqrt {\frac {1}{\mathrm {n-1}}\sum \nolimits _{\mathrm {K=1}}^{\mathrm {n=4}} \left ({{\mathrm {(Actual-Estimated)}}_{\mathrm {k}}\mathrm {-Bias} }\right)^{2}}\tag{2}\end{equation*}

**FIGURE 12.**
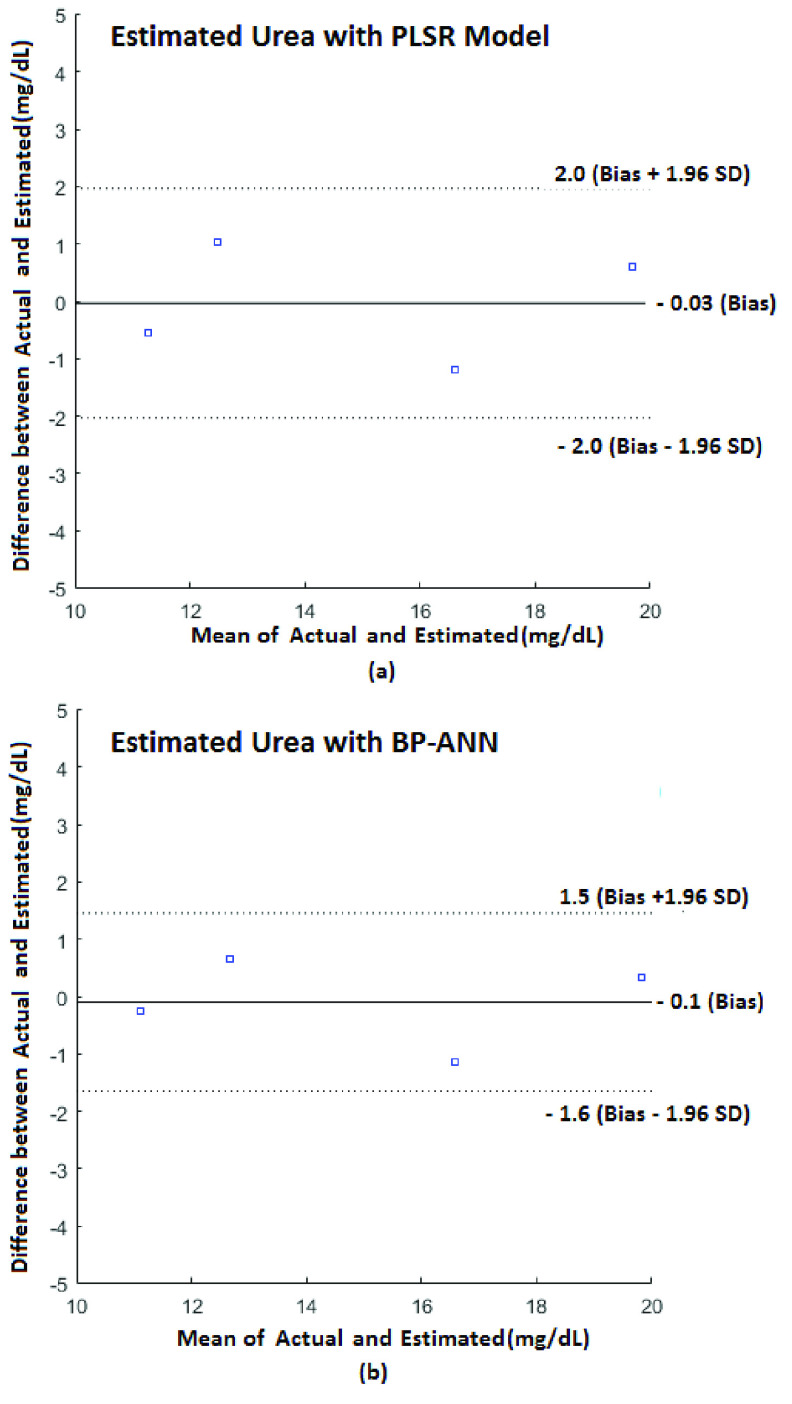
Bland –Altman analysis for blood urea a) PLSR Model b) BP-ANN Model.

**FIGURE 13.**
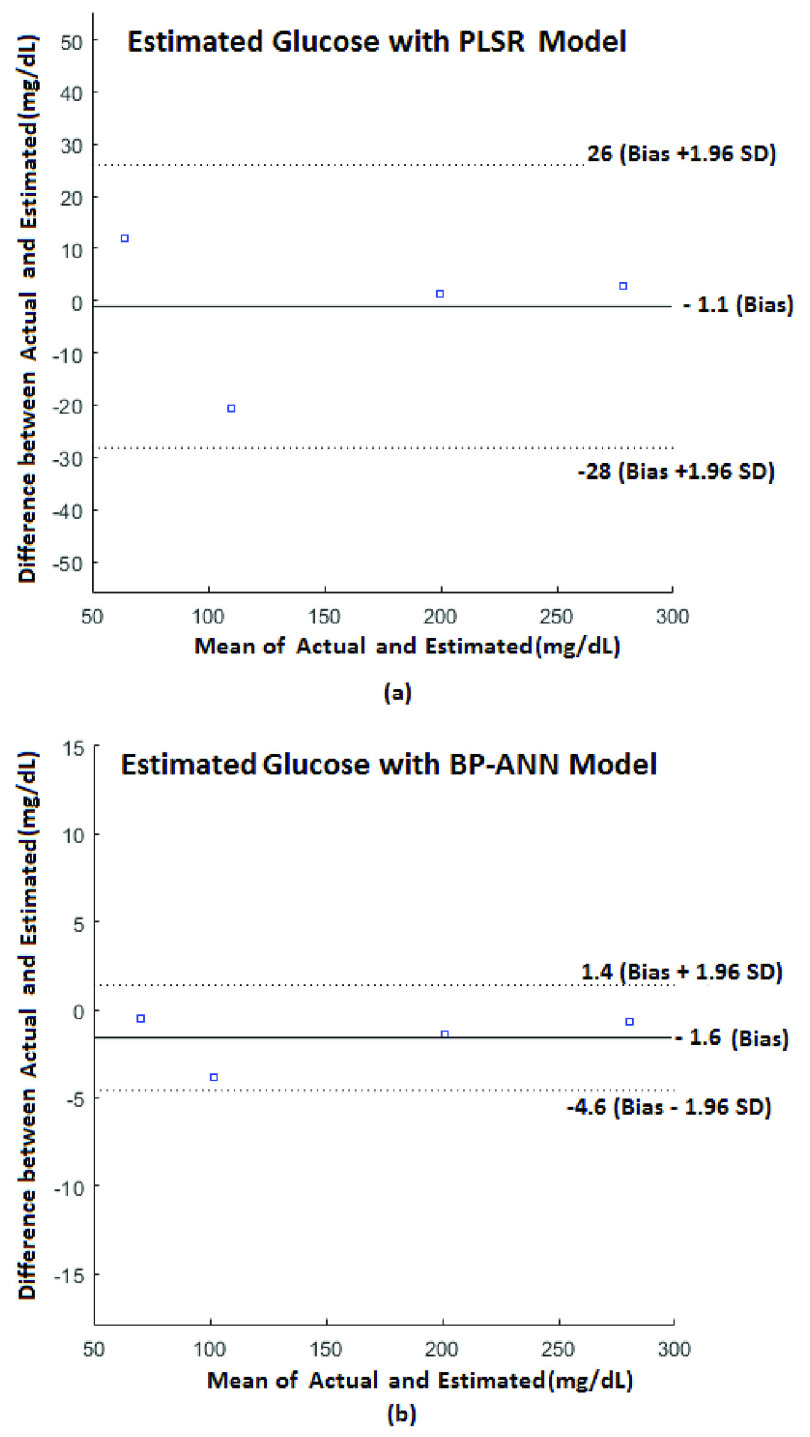
Bland –Altman analysis for blood glucose a) PLSR Model b) BP-ANN Model.

From figure 12, it can be observed that the mean of the difference of estimated and actual value (Bias)/ standard deviation (SD) for estimating blood urea is −0.03 mg/dL/1.02 mg/dL for PLSR Model and −1.0 mg/dL/ 0.79 mg/dL for BP-ANN. Also, from figure 13, Bias/SD for estimating blood glucose is −1.1 mg/dL/13.81 mg/dL for PLSR Model and −1.6 mg/dL/1.52 mg/dL for BP-ANN respectively. From the Bland–Altman plots, it is very clear that most of the readings lie within the limits of agreement and are less spread over for BP-ANN compared to PLSR Model.

#### 2) Clark Error Grid Analysis (CEGA)

**FIGURE 14.**
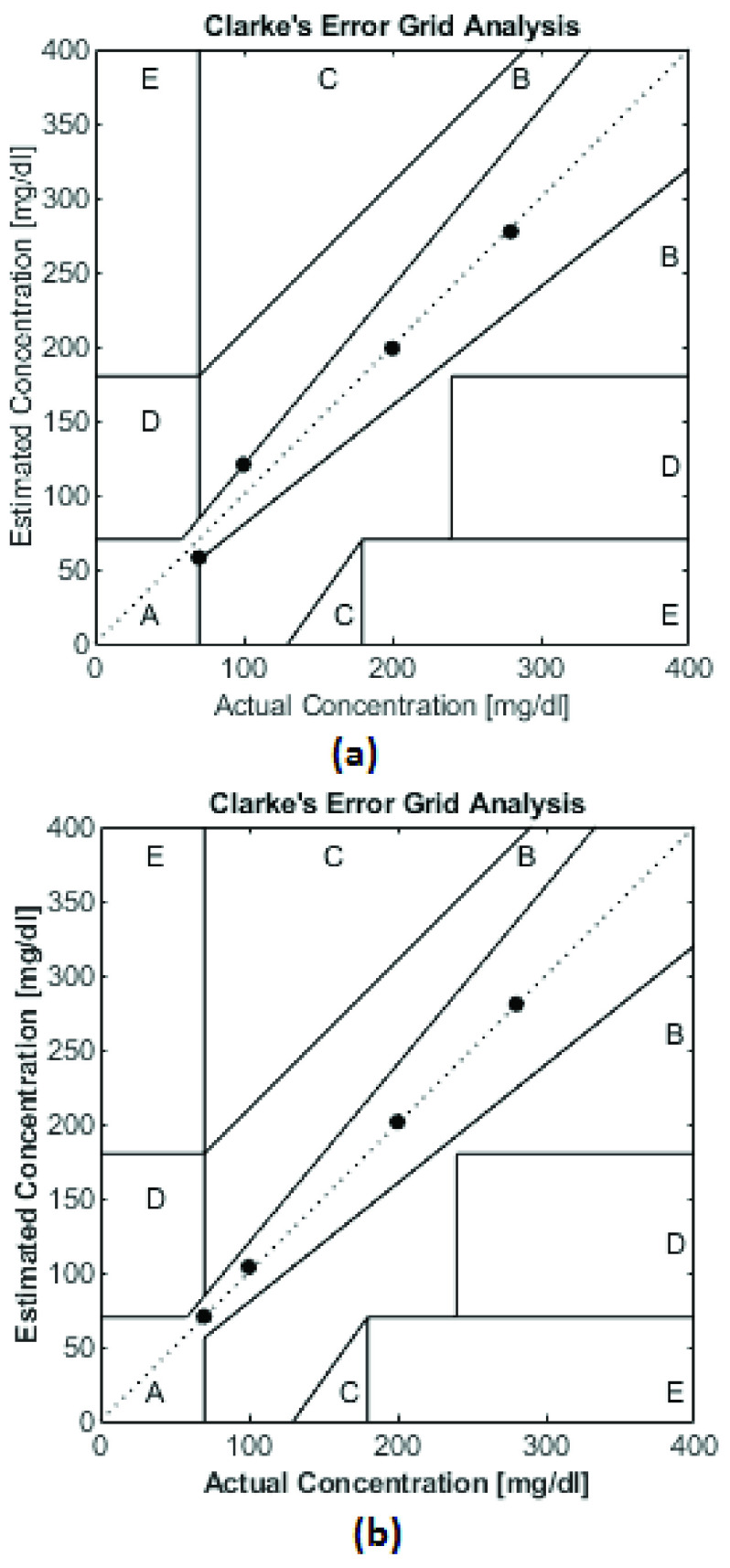
CEGA Plot a) With PLSR b) With BP-ANN.

Any designed system/Instrument for estimating glucose must satisfy the CEGA for clinical acceptance [30]. We have validated our system with CEGA as shown in figure 14(a) with the PLSR model. CEGA plot shows that only two estimated values exactly follow the regression line and two estimated values lie on the edge of Zone A.Zone A signifies the error of less than 20 %. As predicted concentration lie on the borderline, there is uncertainty in accepting this prediction model. The figure 14(b) shows the analysis for the BP-ANN model. It can be seen that a better performance is obtained as all the four estimated values lie in Zone A and exactly follows the best fit regressor line.

### C. Regression Analysis

**FIGURE 15.**
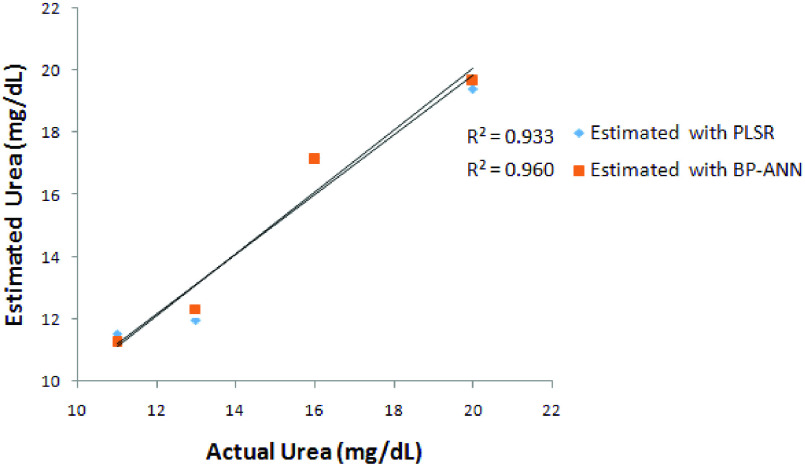
Regression analysis for estimating blood urea with PLSR and BP-ANN model.

**FIGURE 16.**
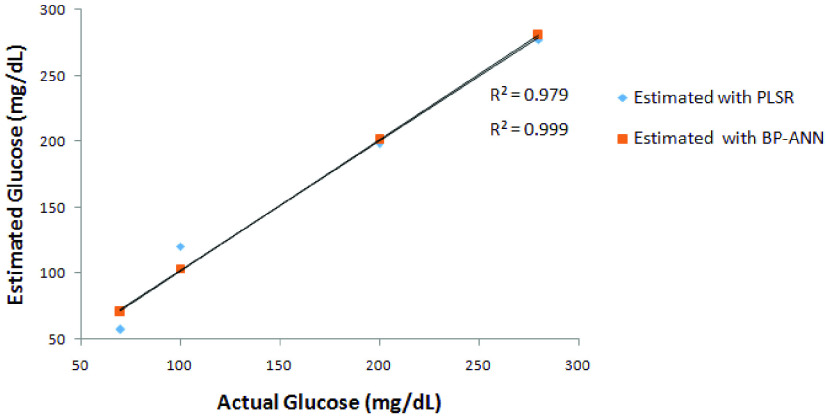
Regression analysis for estimating blood glucose with PLSR and BP-ANN model.

Statistical methods such as Regression analysis are used for finding out the relationships between a dependent variable and one or more independent variables. The regression analysis was done for both urea and glucose with the PLSR model and BP-ANN Model. The regression analysis for urea gives a coefficient of determination (R^2^) of 0.93 for the PLSR model and 0.96 for the BP-ANN Model as shown in figure 15. The same was computed for glucose which gives R^2^ as 0.97 & 0.99 forPLSR and BP-ANN respectively as shown in figure 16.

### D. Accuracy of the System

The system accuracy is calculated using equation 3.

\begin{align*} A=&\left ({1-\frac {\sum {abs\left ({Estimated-Reference }\right)\mathrm {/}} Reference}{No~\mathrm { }of~Subjects} }\right) \\&\ast 100\tag{3}\end{align*}

The accuracy for blood urea estimation using the PLSR model was 94.2% and with the BP-ANN model was 95.96% and for blood glucose estimation using the PLSR model was 90.14% and with the BP-ANN model was 98.65%.

## V. Discussion

Prior to reducing the number of wavelengths points to five, we had designed a PLSR prediction model for the sample which resembled blood over 2500 wavelength points recorded on spectrophotometer V770. This gave excellent accuracy for urea/glucose with RMSE of 0.4/0.5 mg/dL [26]. As our ultimate focus is to make a non-invasive portable system that will be useful for diabetes patients, we have decided to select only five wavelength points in the NIR region which corresponds to spectral features of glucose and urea. The first and second LED wavelength i.e. $2.12~\mu \text{m}$ and $2.24~\mu \text{m}$ are exactly coinciding with urea and glucose signatures and the other three are in the vicinity for both urea and glucose. When PLSR multivariate prediction model was run on these five wavelength points, the prediction error has substantially increased which makes the system unrealizable. With five wavelength points, the PLSR model is not able to find a good correlation between the predictor variables. This led us to explore the ML approach to improve the accuracy of the system. To train the network, PCA components are fed as input to the model. Extracting the main components of the original spectral data for urea and Glucose, the contribution rates of the first three principal components, PC1, PC2, and PC3 were 98.95%,0.92%,0.12%, and the total contribution rate was 99.99%. These three principals components explain most of the spectral feature differences present in glucose and urea. These principal components are then used to train the ML system for predicting glucose and urea. By using BP-ANN we got good accuracy and very low RMSE. This makes the system capable of accurate prediction of glucose. In this paper, we have used the blood resembles samples prepared in the laboratory. Now, we aim to use the actual human tissue for urea and glucose estimation non-invasively.

## VI. Conclusion

The large and growing global burden of Diabetes and CKD needs urgent attention to identify novel monitoring to prevent progressive kidney failure and its complications. Monitoring blood glucose and blood urea in CKD patients non-invasively is very important. To design a non-invasive glucose monitoring system, we have developed a system that estimates blood urea and glucose. The system uses 5-fixed LEDwavelengths in the NIR region. First, the PLSR model was successfully developed in python and executed in NVIDIA Jetson Nano for estimating urea/glucose with an accuracy of 94.2%/90.14%, RMSE of 0.88 mg/dL /12.01 mg/dL, and coefficient of Determination R^2^ = 0.93/0.97 for predicting 4 samples. To obtain better accuracy, the same dataset was trained with BP-ANN to get an accuracy of 95.96%/98.65%, RMSE of 0.69 mg/dL/ 2.06 mg/dL, and coefficient of Determination R^2^ = 0.96/0.99 for predicting the same 4 samples. The PLSR and BP-ANN models for Glucose estimation are validated with CEGA and Bland-Altman Analysis. PLSR model and BP-ANN model for urea estimation were validated with only Bland-Altman analysis. We cannot apply CEGA for urea as it is the gold standard for Glucose estimation. From the analysis, it is clear that BP-ANN outperforms the PLSR model when less spectral information is available.

## Conflicts of Interest

The authors of this article want to declare that there is no conflict between them.
